# Weekly Variations of Intracerebral Hemorrhage Occurrence Among Different Populations: A Cross-Sectional Study

**DOI:** 10.3389/fneur.2021.701929

**Published:** 2021-11-10

**Authors:** Yun-Tao Pu, Ning Yan, En-Yuan Wang, Yan-Yue Wang

**Affiliations:** ^1^Department of Neurology, University-Town Hospital of Chongqing Medical University, Chongqing, China; ^2^Department of Traditional Chinese Medicine, Chongqing University Three Gorges Hospital, Chongqing, China

**Keywords:** intracerebral hemorrhage, weekly, variation, occurrence, region, population

## Abstract

**Background and purpose:** The causes of the higher incidence of intracerebral hemorrhage (ICH) on a given day are unclear. Previous studies have shown that it may vary by region and population. The purpose of this study was to detect weekly variations in ICH occurrence in southwest China and to assess differences in ICH occurrence among different populations.

**Methods:** This hospital-based study included patients with first-onset ICH that occurred from January 1, 2012, to December 31, 2019. The weekly variation in ICH occurrence was analyzed and stratified by sex, age, comorbidities, living habits, and residence.

**Results:** A total of 5,038 patients with first-onset ICH were enrolled. ICH occurrence was higher on Monday [odds ratio (OR), 1.22; 95% CI, 1.09–1.36; *P* < 0.001] and Friday (OR, 1.15; 95% CI, 1.03–1.28; *P* < 0.001) among all patients, and this pattern was consistent with that of men, whereas women showed a higher incidence on Mondays, Saturdays, and Sundays. The increase in the number of ICH events on Monday and Friday was pronounced in the age range of 41–60 years; however, no significant weekly variation in ICH occurrence was observed among other age groups. After stratifying by comorbidities, a significant weekly variation in ICH occurrence was observed in patients with hypertension or diabetes. Smoking and alcohol consumption was associated with a higher incidence of ICH on Friday; otherwise, a Monday excess was observed. The urban population demonstrated a significant weekly variation in ICH occurrence, whereas the rural population did not.

**Conclusions:** Intracerebral hemorrhage occurrence showed weekly variations in southwest China and was significantly affected by sex, age, comorbidities, living habits, and residence. This suggests that weekly variations in ICH occurrence maybe dependent on the region and population.

## Introduction

Although diurnal and seasonal variations in primary intracerebral hemorrhage (ICH) occurrence have been extensively examined worldwide, relatively few studies on weekly variations are available, especially in China. Some studies have reported on variability in ICH occurrence by the day of the week (1, 2); most of them found a higher incidence of ICH on weekdays than on weekends (2–4), and a Monday excess was prominent (5, 6). Wednesday and Thursday were pronounced in ICH occurrence in the study of Jakovljevićrr (2). However, several studies found a higher incidence on weekend after stratifying for sex, age, and drinking (6–8), whereas Shigematsu et al. found no association between weekly variation in ICH occurrence and conventional risk factors (9). In addition, several studies have demonstrated no weekly variation in ICH occurrence (10, 11). The inconsistency of findings has made the conclusions unclear, which perplexed us in making a general prevention strategy for ICH and has also suggested that “days of the week” may not act as conventional risk factors for ICH occurrence (9). However, only a few demographic factors have been analyzed in previous studies, focusing on the stratified analysis of sex and age. Therefore, it may be difficult to demonstrate the discrepancy in weekly variation among different populations, and some risk factors for ICH occurrence maybe ignored. O'Donnell et al. found important regional variations in the relative importance of most individual risk factors for stroke, which could contribute to worldwide variations in the incidence of ICH (12). Although conventional risk factors can predict who is more likely to develop ICH, it remains extremely difficult, if not impossible, to predict when a stroke will occur. Guiraud et al. put forward the concept of “stroke-prone state” and speculated that a combination of predisposing genetic or environmental factors and triggers could lead to an imminently high risk of stroke (13). In particular, external triggering factors may play an important role in the acute causation of the disease (14), but very little is known about them until now.

Chongqing is located in southwest China and has a subtropical monsoon humid climate. A report from the National Bureau of Statistics shows that, as of 2019, Chongqing has a permanent population of >30 million. The purpose of this study was to determine whether a weekly variation in ICH occurrence exists in southwest China, and whether sex, age, comorbidities, living habits, and residence have an impact on it. Clarifying the above issues may provide a reference for the formulation of regional and population-based ICH prevention strategies.

## Materials and Methods

### Clinical Data

All data for this hospital-based cross-sectional study were obtained from the Medical Information System of University-Town Hospital of Chongqing Medical University and Chongqing University Three Gorges Hospital, both of which are large comprehensive teaching hospitals. Appropriate institutional review board approval was obtained for this retrospective study. ICH occurrence was based on discharge diagnosis (based on I61 ICD-10 code). Patients with first-onset ICH were screened from January 1, 2012 to December 31, 2019.

Patients were eligible if they were aged 18 years or older and were diagnosed with acute ICH within 24 h after admission by CT and/or MRI. Patients with a history of ICH, subarachnoid hemorrhage, cerebral trauma, and cerebral tumors were excluded. Information on sex, age, date of onset, comorbidities (such as a history of hypertension and diabetes), living habits, and residence was recorded. Hypertension was defined as a self-reported history of hypertension or blood pressure of 140/90 mmHg or higher. Diabetes was defined as a self-reported history of diabetes mellitus or an HbA1c level of 6.5% or higher. Living habits included smoking and drinking. History of smoking was defined as former and current smokers (≥1 cigarette per day) and drinking history as former and current drinkers (at least once per month). Residences were categorized into rural and urban areas, which were determined by the current residential addresses registered in medical records of the subjects.

### Statistical Analysis

Data for the period from January 1, 2012 to December 31, 2019 were pooled and divided into 7 days of the week, and stratified analyses were used to assess the impact of sex, age, comorbidities, living habits, and residence on weekly variation in ICH occurrence. Subjects were stratified into three categories according to their age: young adults (≤40 years), middle-aged people (41–60 years), and older people (>60 years).

All statistical analyses were performed using IBM SPSS (version 22.0; IBM Corp., Armonk, NY, USA). Mean age was expressed as mean (SD), while other variables were presented as counts (percentages). Assuming that the day of the week has no effect on ICH occurrence, a roughly uniform distribution should be observed. Therefore, the average for each day of the week was taken as the expected value. Data were statistically analyzed with χ^2^ tests for goodness of fit to the null model of equal distribution of ICH to evaluate the weekly variations in ICH onset. To estimate the odds ratio (OR) with a 95% CI of ICH occurring on a specific day of the week, the observed number was compared with the average number for each day of the week. Statistical significance was set at *P* < 0.05.

## Results

**Figure 1:**
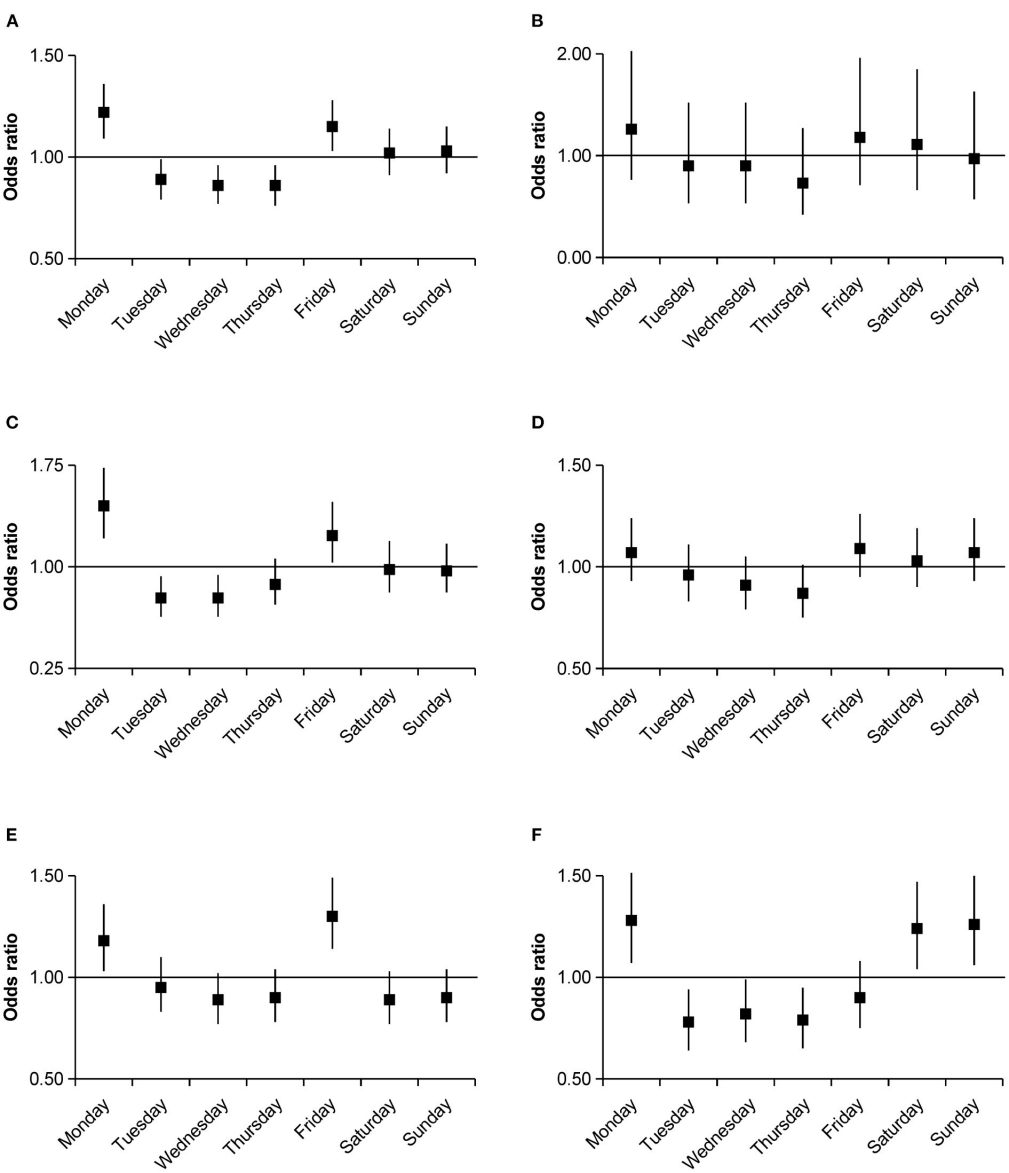
Odds ratios with 95% confidence intervals for weekly variations of intracerebral hemorrhage occurrence compared with the average numbers. **(A)** Total group. **(B)** ≤40 years group. **(C)** 41–60 years group. **(D)** >60 years group. **(E)** Men group. **(F)** Women group.

A total of 5,038 subjects with first-onset ICH and a mean age of 62.2 ± 12.8 years were included. Their demographic characteristics, such as sex, age, comorbidities, living habits, and residence, are presented in Table 1. Overall, ICH occurrence was higher on Monday (OR, 1.22; 95% CI, 1.09–1.36; *P* < 0.001) and Friday (OR, 1.15; 95% CI, 1.03–1.28; *P* < 0.001) among all subjects (Figure 1).

**Table 1:** Characteristics of patients with first-onset intracerebral hemorrhage.

	**Intracerebral hemorrhage**
	**(*n* = 5,038)**
**Demographics**	
Age, y, mean (SD)	62.2 (12.8)
**Age distribution**, ***n*** **(%)**	
≤ 40 y	231 (4.6)
41–60 y	1,789 (35.5)
>60 y	3,018 (59.9)
**Sex**, ***n*** **(%)**	
Men	3,141 (62.4)
Women	1,897 (37.7)
**Comorbidities**, ***n*** **(%)**	
Hypertension	3,221 (63.9)
Diabetes	412 (8.2)
**Living habits**, ***n*** **(%)**	
Drinking	1,374 (27.3)
Smoking	1,546 (30.7)
**Residence**, ***n*** **(%)**	
Urban	3,409 (67.7)
Rural	1,629 (32.3)

*SD indicates standard deviation. Data are n (%) or mean (SD)*.

**Figure 2:**
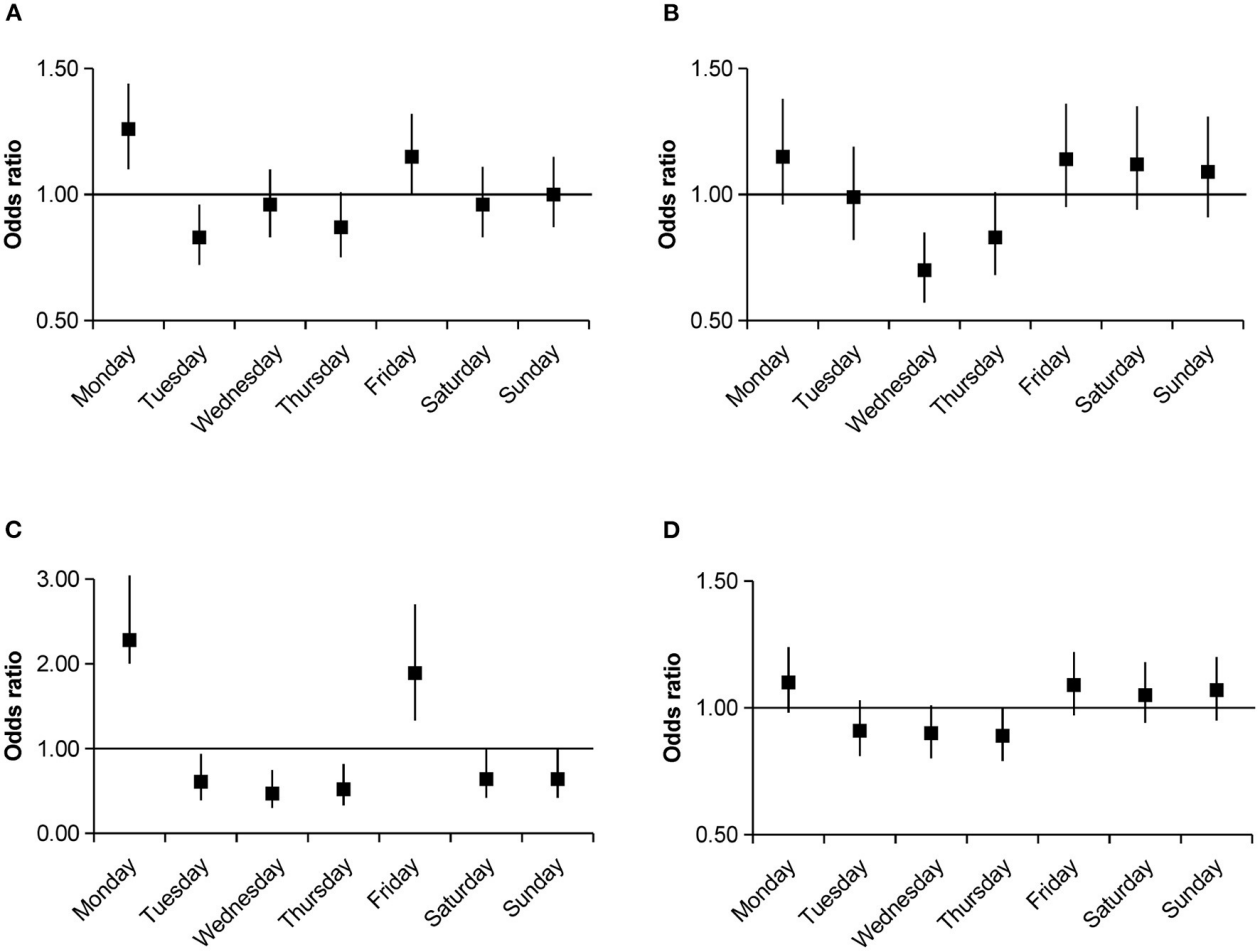
Odds ratios with 95% confidence intervals for weekly variations of intracerebral hemorrhage occurrence stratified by hypertension and diabetes group compared with the average numbers. **(A)** Hypertension group. **(B)** Non-hypertension group. **(C)** Diabetes group. **(D)** Non-diabetes group.

**Figure 3:**
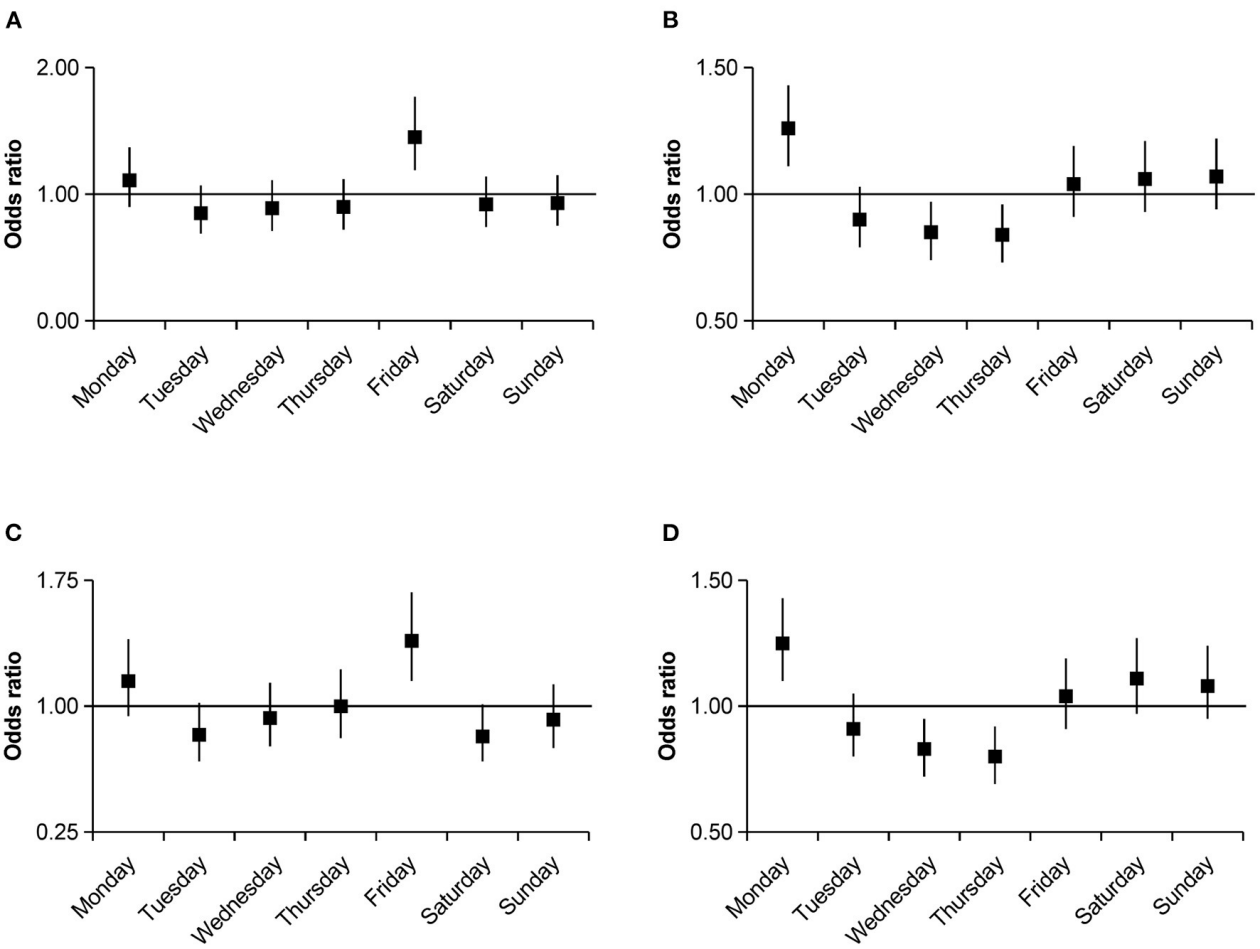
Odds ratios with 95% confidence intervals for weekly variations of intracerebral hemorrhage occurrence stratified by drinking and smoking group compared with the average numbers. **(A)** Drinking group. **(B)** Non-drinking group. **(C)** Smoking group. **(D)** Non-smoking group.

**Figure 4:**
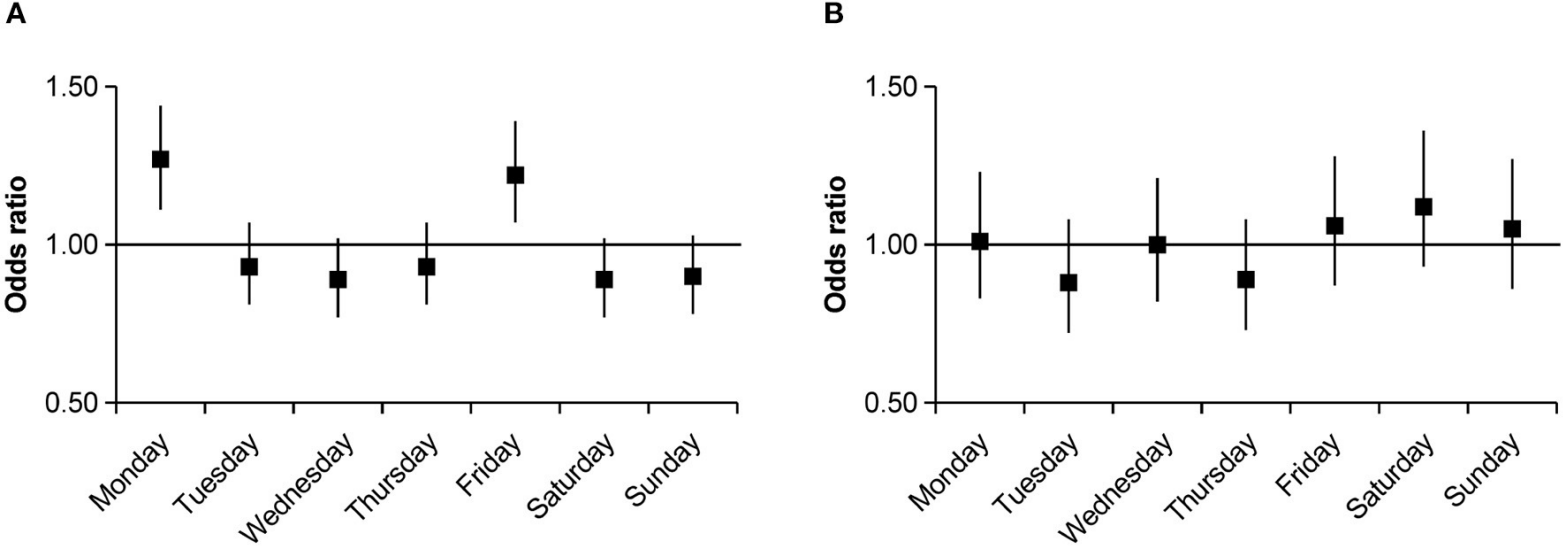
Odds ratios with 95% confidence intervals for weekly variations of intracerebral hemorrhage occurrence stratified by residence group compared with the average numbers. **(A)** Urban group. **(B)** Rural group.

After further stratification by age, an increase in the number of ICH events on Monday (OR, 1.45; 95% CI, 1.21–1.73; *P* < 0.001) and Friday (OR, 1.23; 95% CI, 1.03–1.48; *P* = 0.024) was pronounced in the 41–60 age group, whereas no significant weekly variation in ICH occurrence was observed among other age strata. Further analysis by sex group showed a higher ICH occurrence on Monday (OR, 1.18; 95% CI, 1.03–1.36; *P* = 0.016) and Friday (OR, 1.3; 95% CI, 1.14–1.49; *P* < 0.001) in men; however, women had a higher incidence on Monday (OR, 1.28; 95% CI, 1.07–1.52; *P* = 0.006), Saturday (OR, 1.24; 95% CI, 1.04–1.47; *P* = 0.018), and Sunday (OR, 1.26; 95% CI, 1.06–1.5; *P* = 0.01). After stratifying by comorbidities, we only observed a significant weekly variation in ICH occurrence in patients with comorbidities of hypertension or diabetes. In the hypertension group, there were clear incidence peaks on Monday (OR, 1.26; 95% CI, 1.1–1.44; *P* = 0.001) and Friday (OR, 1.15; 95% CI, 1–1.32; *P* = 0.044); similar peaks were observed in the diabetes group (Monday OR, 2.28; 95% CI, 2–3.98; *P* < 0.001; Friday OR, 1.89; 95% CI, 1.33–2.7; *P* < 0.001; Figure 2). Smoking (OR, 1.39; 95% CI, 1.15–1.68; *P* = 0.001) and drinking (OR, 1.45; 95% CI, 1.19–1.77; *P* < 0.001) were associated with a higher incidence of ICH on Friday. In the non-smoking (OR, 1.25; 95% CI, 1.1–1.43; *P* = 0.001) and non-drinking groups (OR, 1.26; 95% CI, 1.11–1.43; *P* < 0.001), a Monday excess was observed (Figure 3). In the residential strata, the urban population demonstrated significant peaks on Monday (OR, 1.27; 95% CI, 1.11–1.44; *P* < 0.001) and Friday (OR, 1.22; 95% CI, 1.07–1.39; *P* = 0.003). However, no particular weekly pattern of ICH occurrence was observed in the rural population (Figure 4).

## Discussion

The present study showed a weekly variation in ICH occurrence in southwest China, and it was significantly affected by sex, age, comorbidities, living habits, and residence. Overall, a higher incidence of ICH was associated with Mondays and Fridays among all patients. However, stratified analyses of demographic characteristics and risk factors demonstrated significant differences in weekly distributions of ICH occurrence.

This study demonstrated a higher incidence of ICH on Monday, which is consistent with previous studies (5, 6). The majority of researchers believe that this may be the result of sudden changes in physical and mental activities during the transition from weekends to weekdays (Sunday–Monday). Although Cranford et al. believed that the mental stress on Monday was worse than that on other days of the week (15). Ryan et al. found no significant difference in mental stress between Mondays and any other workday except Friday (16). However, the hypothesis of a sudden change in mental activity due to the transition from weekends to weekdays was more generally accepted (17). The sudden change in mental activity may largely contribute to the “Blue Monday” phenomenon (15, 18). Although an increase in the number of ICH events on Friday was rarely observed in previous studies (2). The present study demonstrated a high incidence of ICH on Friday, which may be attributed to several potential reasons. First, O'Donnell et al. found that psychosocial factors are closely related to stroke incidence in China (12). It is well known that Friday is the last workday, and work reviews are usually performed on Friday, which may increase mental stress on Friday. In addition, several studies have demonstrated that not only negative emotions but also sudden changes in positive emotions can trigger ICH (13, 19). For most working subjects, the positive weekend emotions begin on the Friday afternoon (16). Finally, recreational activities beginning after work on Friday for many working adults, such as overeating and excessive drinking, are good candidates as triggering factors of ICH occurrence (7).

After stratification by demographic characteristics and risk factors, men had a similar peak incidence of ICH as the overall population. In addition to Monday, women demonstrated an increase in ICH incidence on weekends (1, 3, 7). This phenomenon may be explained, at least in part, by the very different traditional sex roles in Chinese family life. Women may take more responsibility for household chores and raising children, which may keep them in a continuous working pattern throughout the week, and the combined stress from work and household chores may deteriorate their health (8, 20). In addition, the increased incidence of ICH on weekends may suggest that a sudden shift in the day-to-day activity pattern may be a good candidate as a triggering mechanism of ICH (7, 21, 22).

The weekly variation in ICH occurrence was significant only among subjects aged 41–60 years. This phenomenon is probably related to the fact that middle-aged subjects in China might have to face more stress in the work environment and bear more responsibility in the family (23, 24). In China, other age strata, particularly those over 60 years old, faceless mental stress because most of them have retired. Stone et al. found that subjects aged 30–59 experience the greatest increase in negative feelings from Sunday to Monday, while the mood gap between Sunday and Monday declines as age increases (17). Therefore, less mental stress and a more stable lifestyle in retired subjects may contribute to the less pronounced weekly variation in ICH occurrence (4, 14).

This study demonstrated that regardless of the presence or absence of hypertension or diabetes, the peak incidence of ICH was similar to that of the overall population, which is consistent with findings of other studies (8, 9). However, a significant weekly variation in ICH occurrence was observed in patients with comorbidities of hypertension or diabetes, which was inconsistent with the results of previous studies. It is well known that hypertension and diabetes are common and important risk factors for ICH (12), which may increase vulnerability to ICH occurrence (25). This may be the reason why only patients with comorbidities had a significant weekly variation in ICH occurrence. Therefore, we speculate that subjects with higher vulnerability are more predisposed to developing ICH when exposed to the same triggering factors (13).

Smoking and drinking were found associated with a higher incidence of ICH on Friday, whereas, in non-smoking and non-drinking groups, a Monday excess was observed. Smoking and drinking are well-established risk factors for ICH, and therefore may increase the vulnerability to ICH occurrence (12). However, this fact does not explain the peak incidence of ICH on Friday. It has been reported that later time on Friday is the start of free time for working adults (16), and drinking is likely to be one of the main recreational activities (7). As drinking may be one of the triggering factors for a higher incidence of ICH on Friday, this is consistent with our hypothesis. Furthermore, drinking and smoking are more likely to co-exist in the Chinese population, especially among men (26, 27). All the abovementioned factors may be the reason why drinking and smoking subjects are more likely to develop ICH on Friday.

After stratification by residence, the urban population demonstrated a significant weekly variation in ICH occurrence, whereas the rural population did not. The rural population is usually engaged in traditional agricultural production, and there may not be a difference between weekdays and weekends in their lifestyle (28). Therefore, the rural population may not suffer weekly variations in physical activity and mental stress, which is consistent with the characteristics of the retired population. Therefore, we can speculate that populations without significant weekly variation in lifestyle have a roughly uniform distribution of ICH incidence throughout the week (14).

## Strengths and Limitations

This study has several strengths. First, there are few known studies in China on the addressed topic; therefore, this study provides references for region-specific ICH occurrence. Furthermore, compared with previous studies, more characteristics and risk factors were included in the analysis as population variables in this study. Finally, this study included residence in the analysis, which is a local characteristic variable in China.

However, this study also has a few limitations. First, patients who did not visit the hospital or visited other hospitals may have been missed. As a result, obtained findings may not be fully representative of the characteristics of ICH occurrence in the local region. However, the sample size was large enough to overcome this deficiency to some extent. Furthermore, more specific information, such as disease duration, medication, current status, and organ damage, was lacking in patients with hypertension or diabetes. This may preclude in-depth exploration of the impact of comorbidities on ICH occurrence and a more accurate assessment of the vulnerable factors. In addition, we could neither determine the duration of drinking and smoking nor quantify the amount of alcohol or cigarette smoking, which makes further study impossible. Moreover, the weekly changes in mood of the patients and activity quantitative scores were not evaluated in this retrospective study; therefore, it is not possible to directly conclude the influence of external stimulus factors on the weekly rhythm of ICH. Many of the discussed explanations were based on the general characteristics of the specific population and should be confirmed by future prospective studies. Finally, the information of the residences of the patients may be inaccurate or not sufficiently detailed, and patients whose residences were in a state of constant change were not excluded. Although these patients cannot be grouped accurately, we believe that the proportion of these patients was very small and has little impact on obtained results.

## Conclusion

This study showed a weekly variation in ICH occurrence in southwest China and its dependence on region and population. Due to the differences in working style and living habits, the highest incidence of ICH may occur on any day of the week in different populations. We should not only focus on whether a given day of the week has a higher incidence of ICH; perhaps, more attention should be paid to the factors affecting the incidence of ICH on a given day. This study provides evidence that regional and population characteristics should be taken into account when formulating strategies for ICH prevention.

## Data Availability Statement

The raw data supporting the conclusions of this article will be made available by the authors, without undue reservation.

## Ethics Statement

The studies involving human participants were reviewed and approved by the Ethics Committee of University-Town Hospital of Chongqing Medical University and Chongqing University Three Gorges Hospital. Written informed consent for participation was not required for this study in accordance with the national legislation and the institutional requirements.

## Author Contributions

Y-TP and Y-YW designed the research. Y-TP, Y-YW, E-YW, and NY performed data collection and statistical analysis. Y-TP wrote the first draft of the manuscript. All authors have read and approved the manuscript.

## Conflict of Interest

The authors declare that the research was conducted in the absence of any commercial or financial relationships that could be construed as a potential conflict of interest.

## Publisher's Note

All claims expressed in this article are solely those of the authors and do not necessarily represent those of their affiliated organizations, or those of the publisher, the editors and the reviewers. Any product that may be evaluated in this article, or claim that may be made by its manufacturer, is not guaranteed or endorsed by the publisher.
